# High Genetic Diversity among Community-Associated *Staphylococcus aureus* in Europe: Results from a Multicenter Study

**DOI:** 10.1371/journal.pone.0034768

**Published:** 2012-04-27

**Authors:** Joana Rolo, Maria Miragaia, Agata Turlej-Rogacka, Joanna Empel, Ons Bouchami, Nuno A. Faria, Ana Tavares, Waleria Hryniewicz, Ad C. Fluit, Hermínia de Lencastre, the CONCORD Working Group

**Affiliations:** 1 Laboratory of Molecular Genetics, Instituto de Tecnologia Química e Biológica, Universidade Nova de Lisboa, Oeiras, Portugal; 2 National Medicines Institute, Division of Clinical Microbiology and Infection Prevention, Warsaw, Poland; 3 Laboratory of the National Bone Marrow Transplantation Centre, Bab Saadoun, Tunis, Tunisia; 4 Department of Medical Microbiology, University Medical Center Utrecht, Utrecht, The Netherlands; 5 Laboratory of Microbiology, The Rockefeller University, New York, New York, United States of America; National Institutes of Health, United States of America

## Abstract

**Background:**

Several studies have addressed the epidemiology of community-associated *Staphylococcus aureus* (CA-SA) in Europe; nonetheless, a comprehensive perspective remains unclear. In this study, we aimed to describe the population structure of CA-SA and to shed light on the origin of methicillin-resistant *S. aureus* (MRSA) in this continent.

**Methods and Findings:**

A total of 568 colonization and infection isolates, comprising both MRSA and methicillin-susceptible *S. aureus* (MSSA), were recovered in 16 European countries, from community and community-onset infections. The genetic background of isolates was characterized by molecular typing techniques (*spa* typing, pulsed-field gel electrophoresis and multilocus sequence typing) and the presence of PVL and ACME was tested by PCR. MRSA were further characterized by SCC*mec* typing. We found that 59% of all isolates were associated with community-associated clones. Most MRSA were related with USA300 (ST8-IVa and variants) (40%), followed by the European clone (ST80-IVc and derivatives) (28%) and the Taiwan clone (ST59-IVa and related clonal types) (15%). A total of 83% of MRSA carried Panton-Valentine leukocidin (PVL) and 14% carried the arginine catabolic mobile element (ACME). Surprisingly, we found a high genetic diversity among MRSA clonal types (ST-SCC*mec*), Simpson’s index of diversity = 0.852 (0.788–0.916). Specifically, about half of the isolates carried novel associations between genetic background and SCC*mec*. Analysis by BURP showed that some CA-MSSA and CA-MRSA isolates were highly related, suggesting a probable local acquisition/loss of SCC*mec*.

**Conclusions:**

Our results imply that CA-MRSA origin, epidemiology and population structure in Europe is very dissimilar from that of USA.

## Introduction

*Staphylococcus aureus*, particularly methicillin-resistant *S. aureus* (MRSA) is one of the most important nosocomial pathogens. Resistance to methicillin is conferred by the *mecA* gene, which is carried within a mobile genetic element called staphylococcal cassette chromosome *mec* (SCC*mec*) [1]. So far, eleven major structural types of SCC*mec* have been described [2], [3], [4], [5].

For several decades, MRSA was confined to hospitals, but in the early 1990s, infections in healthy individuals emerged among Aborigines’ communities in Australia [6]. However, clinical significance was attributed to these strains only some years later when four children with no previous hospital contact died in the USA due to MRSA infection [7]. Although the very first outbreaks of serious infections in the USA were caused by the USA400 clone (ST1-IVa) [7], USA300 (ST8-IVa) rapidly become the dominant community-associated MRSA (CA-MRSA) clone in the USA [8]. Molecular typing studies showed that CA-MRSA differs from hospital-associated MRSA (HA-MRSA) [9]: they belong to distinct genetic lineages, usually carry smaller SCC*mec* cassettes and specific virulence factors, such as PVL (Panton-Valentine leukocidin) and ACME (arginine catabolic mobile element). CA-MRSA were also shown to have higher expression levels of toxins, PSMs (phenol soluble modulins) and hemolysins [10], [11], suggesting that these isolates are more virulent than HA-MRSA.

Previous studies have shown that USA300 is extremely widespread in the USA [8], [12], whereas CA-MRSA infections in other parts of the world are generally caused by other clones: the Southwest-Pacific clone (ST30-IVc) and the Queensland clone (ST93-IVa) [13], [14] in Australia and New Zealand; the Taiwan clone (ST59-IVa, ST59-V) and USA700 (ST72-IVc) in Asia [15], ; ST88-IV in Africa [18] and the European clone (ST80-IVc) in Europe [19]. The dominant CA-MRSA clones in different European countries have been identified [20], [21], [22], [23], [24], [25], [26], [27], [28], [29], [30]. Nonetheless, the comprehensive population structure of CA-MRSA and MSSA has never been assessed in Europe. In this study we aim to identify the main CA-MRSA and MSSA clonal lineages in Europe, to determine its population structure and to try to shed some light on the origin of CA-MRSA in this continent.

## Methods

### Ethical Statement

Isolates were obtained as part of routine diagnostic testing and were analyzed anonymously and the isolates, not humans, were studied. All data was collected in accordance with the European Parliament and Council decision for the epidemiological surveillance and control of communicable disease in the European community [31], [32]. Ethical approval and informed consent were thus not required.

### Bacterial Collection

The collection analyzed in this study was not part of a structured survey with pre-defined criteria of isolate collection. It was a convenience sample composed of 568 *S. aureus* isolates obtained from infection (74%) and colonization (17%) of patients attending health-care centers and hospitals, collected within 48 hours of hospitalization. The isolates were distributed among 16 of the most populous countries in Europe, including The Czech Republic (75 isolates), Spain (52), The Netherlands (49), Greece (45), United Kingdom (42), Sweden (41), Hungary (40), Bulgaria (37), Denmark (37), France (34), Poland (26), Romania (24), Portugal (22), Finland (20), Slovakia (17) and Italy (7). The isolates were obtained between 2000 and 2010, but the majority have been collected between 2007 and 2010 (62%). For 9%, no information on collection year was available. All isolates were accompanied by a questionnaire with clinical data and with questions formulated to ascertain their origin. The isolates were grouped according with their presumptive origin as suggested previously [8]: isolates were considered as community-associated *S. aureus* (CA-SA) when they were collected from persons that had no previous contact with the hospital one year before sampling or any of the assessed risk factors for MRSA carriage and were obtained in the first 48 hours of hospitalization (Center for Disease Control criteria); isolates were considered to be community onset *S. aureus* (CO-SA) when they were collected from persons with at least one risk factor for health-care contact although they were obtained within the first 48 hours of hospitalization. For some of the isolates no information was available regarding several of the assessed risk factors for hospital contact. In these cases isolates were considered as having a community-onset origin.

### Patient Population

The patient population included 231 females and 264 males; for 73 patients, no information was available regarding gender. A total of 122 children (0–18 years), 285 adults (19–64) and 119 elderly (>65) were included in the study; no information was available regarding age for 42 patients. The clinical diagnosis for a great part of the patients (52%) was skin and soft tissue infections (SSTIs), bacteremia or septicemia (6%), endocarditis (5%), pneumonia (4%) and urinary tract infections (3%). For 30%, no information regarding clinical diagnosis was available.

### *S. aureus* Isolation and Cultivation

*S. aureus* were isolated by standard techniques and identified by Vitek (Vitek2, bioMérieux, Marcy L’Etoile, France) or phenotypic methods in participating institutions. Species identification was confirmed by growth on mannitol salt agar (MSA, Difco, BBL, Becton Dickinson, Franklin Lakes, New Jersey, USA) and by testing coagulase production by Staphytec Plus assay (Oxoid, Cambridge, United Kingdom).

### Antimicrobial Susceptibility Testing

All isolates were tested by Vitek (Vitek 2, bioMérieux, Marcy L’Etoile, France, cards P563, 580, 592, 619), by disk diffusion or broth microdilution methods, according to the Clinical and Laboratory Standards Institute recommendations [33] in the collaborating centers against a panel of 22 antimicrobial agents, including: oxacillin, benzylpenicillin, cefoxitin, vancomycin, teicoplanin, linezolid, gentamicin, tobramycin, norfloxacin, ciprofloxacin, levofloxacin, moxifloxacin, erythromycin, clindamycin, quinupristin/dalfopristin, tetracycline, rifampicin, fosfomycin, mupirocin, fusidic acid, trimethoprim/sulfamethoxazole, nitrofurantoin. For 157 isolates (28%) the antibiogram was not performed. Multiresistance was defined as resistance to three or more classes of antimicrobial agents. The rates of resistance and multidrug resistance of CA epidemic clones were calculated considering as denominator the total number of isolates with antimicrobial susceptibility testing data.

### Detection of the *mecA* and Panton-Valentine Leukocidin (PVL) Genes

The *mecA* and *lukS-PV*, *lukF-PV* genes (which encode PVL) were detected as described before [34], [35].

### SCC*mec* Typing

The structure of the staphylococcal cassette chromosome *mec* (SCC*mec*) was determined by the updated multiplex PCR strategy developed by Milheiriço et al [36]. The subtypes of SCC*mec* type IV were determined by multiplex PCR as previously described [37]. In case of ambiguous results this characterization was further complemented by amplification of the *mec* complex and *ccr* complexes by PCR [9], [38], [39] and by sequencing an internal region of the *ccrB* gene [39], [40]. SCC*mec* was classified according to the guidelines proposed by the International Working Group on the Classification of SCC elements [2]. A SCC*mec* was considered non-typable (NT) when it was not possible to ascertain a class of *mec* complex and/or a type of *ccr*.

### *spa* Typing

*spa* typing was performed as previously described [41] and *spa* types were attributed using the RIDOM web server (http://spaserver.ridom.de/). The BURP algorithm was used to define *spa* clonal complexes (*spa*-CC) [42]. The *spa* server was used as well to predict sequence types (STs).

### Pulsed-field Gel Electrophoresis (PFGE)

PFGE was performed: 1) to predict STs, when a single ST could not be inferred from the *spa* type; 2) to discern the most closely related clonal type among MSSA strains, when this could not be deduced from ST (e.g. ST8-MSSA, could be related to USA300 clone or Irish clone (ST8-II)). Total DNA was restricted with SmaI and the resulting fragments were separated by PFGE as described before [43]. The SmaI restriction band assignments were manually curated after automatically band detection, using the Bionumerics software (Applied Maths, Saint-Martens-Latem, Belgium). For band patterns comparison with reference strains belonging to epidemic clones of nosocomial and community origin, the following settings were used: optimization of 0.50% and a tolerance of 1.25%. PFGE types were defined by groups formed at 80% Dice similarity cutoff on a dendrogram constructed by the unweigthed pair group method using average linkage (UPGMA), as previously described [40].

### Multilocus Sequence Typing (MLST)

MLST was performed when a sequence type (ST) could not be inferred from the *spa* type in the *spa* server (http://spaserver.ridom.de/) or from the literature. The genetic background of the isolates was determined by MLST [44]. STs were attributed by submitting the DNA sequences obtained to the online MLST database (http://www.mlst.net/). The goeBURST algorithm was used to assign MLST clonal complexes, CC (http://goeburst.phyloviz.net). This analysis was performed on October 20^th^, 2011.

### ACME Detection

The two loci (*arc and opp3*) that compose ACME-I in USA300 strain FPR3757, were amplified by PCR as previously suggested [45]. This element was typed according with its structure: type I (*arc* and *opp3* operons), type II (*arc* operon only) and type III (*opp3* operon only) [45], [46]. ACME was detected only in the isolates that were related with community-associated clones (see below).

### Definition of CA-MSSA/CA-MRSA Epidemic Clones and CA-MRSA/CA-MSSA Clone-related

Community-associated (CA), hospital-associated (HA) and live stock-associated (LA) epidemic clones were defined as clones (MSSA:ST; MRSA:ST-SCC*mec*) previously described to cause infections in several different geographic locations and that have originated in the community, hospital and animals, respectively [47]. In this study, MSSA clones were defined by its ST, *spa* type, PFGE type (when necessary), ACME and PVL. Each MRSA clone was defined based on the association of ST and SCC*mec* type as previously proposed [44], as well as by *spa* type, ACME and PVL. A clone was considered to be related to other if they shared all these characteristics except one. For ST, only single locus variants were considered related. When the molecular characteristics of an isolate did not fulfill these criteria, the isolate was considered sporadic.

### Assessment of Genetic Diversity

The degree of genetic diversity was assessed by the Simpson’s index of diversity (SID), using a confidence interval of 95% [48]. The SID was estimated by the combination of the results obtained by *spa* typing, PVL and ACME determination (each different combination was considered a “type” or a “species”).

## Results

### Population Structure of *S. aureus* Isolated from the Community-associated Setting

A total of 246 isolates (43%) out of the 568 isolates analyzed in this study were isolated from subjects with no recent hospital contact (CDC criteria for CA-SA). The combination of the results obtained by all the typing methods used showed that the majority (145 isolates, 59%) was related to epidemic community-associated (CA) clones, while 67 (27%) were related to hospital-associated (HA) clones and 32 (13%) were sporadic [47]. In addition, we recovered two MSSA isolates (1%) belonging to livestock-associated clones or related (ST398 and its SLV, ST291) (Table 1).

**Table 1:** Distribution of the isolates analyzed in this study.

Isolate collection/risk factor for hospital contact	Total no of isolates	Infection/colonization (no of isolates)	MSSA/MRSA	Type of epidemic clones (no of isolates,%)
<48 h with risk factor (CO-MRSA/MSSA)	322	225/46*	172/150	CA (193, 60%); HA (85, 26%); LA (2, 1%); Sporadic (42, 13%)
<48 h with no risk factors (CA-MRSA/MSSA)	246	198/48	107/139	CA (145, 59%); HA (67, 13%); LA (2, 1%); Sporadic (32, 13%)

* for 51 isolates no information was available regarding this specific question.

Of the 67 *S. aureus* isolates belonging to HA clones, 43 were MRSA and were related with EMRSA15 (ST22-IVh) (19 isolates, 46%), EMRSA-3 (ST5-I) (11 isolates, 24%), and Pediatric (ST5-VI/IV) (six isolates, 15%) clones. The remaining seven isolates belonged to New York Japan (ST5-II, 3 isolates), Berlin (ST45-IV), Brazilian (ST239-III), ST111-I, and ST125-V (one isolate each). The 24 MSSA isolates belonged to ST45 (10 isolates, 43%), ST5 (seven isolates, 26%), ST22 (five isolates, 22%) and ST36 (two isolates, 9%).

Of the 145 isolates belonging to CA epidemic clones, 58% (84 isolates) were MRSA and belonged or were related to USA300 (29 isolates, 37%) and the European epidemic clones (28 isolates, 36%). The remaining isolates belonged to: the Taiwan or related clones, ST59-V, ST59-IVa (four isolates each, 11%), and a ST59 SLV, ST375-IVa (three isolates); ST772-V (five isolates); ST1-IVa (three isolates); ST97-IVa (two isolates); ST15-IVa, ST30-IVa, ST30-IVc, ST72-IVc, ST93-IVa, and ST188-IVa (one isolate each).

Noteworthy, we observed that a considerable part of the isolates related to epidemic CA-MRSA clones lacked one or more of their typical characteristics, namely the presence of PVL and/or ACME or did not belong to a particular *spa* type or ST (Table 2 and Information S1). For example, only 11 isolates out of the 29 isolates related to USA300 had all the molecular characteristics of this clone (ST8-IVa, t008, PVL+ and ACME-I) [12] and 23 out of 28 isolates had all the characteristics of the European clone (ST80-IVc, t044, PVL+) [49]. Overall a high genetic diversity of MRSA clones, as determined by the combination of the results obtained by *spa* typing, PVL and ACME determination, was observed [SID = 0.852 (0.788–0.916)].

**Table 2:** Molecular characteristics of the 338 CA and CO-*S. aureus* isolates analyzed in this study that belonged to epidemic CA clones or related.

Genetic background (no of isolates; %)	Sequence types (MLST)	SCC*mec* types	*spa* types	PVL (no positive isolates/total isolates)	ACME (no positive isolates/total isolates)
**CC8 (92; 27)**	ST8, ST72, ST931, ST939	IVa, IVc, IVd, IVg, IVnt, V, VI, NT	t008, t024, t064, t148, t121, t324, t664, t791,t1189, t1578, t5160, t1705	59/72	40/72
	ST8, ST72	MSSA	t008, t024, t126, t148, t3682, t5896	2/20	1/20
**CC15 (57; 17)**	ST1, ST15, T188,ST772, ST1835	IVa, V, NT	t084, t127, t189, t345, t657, t1381, t4915	8/13	–
	ST1, ST15, ST772, ST1867	MSSA	t084, t085, t121, t127, t184, t273, t346, t368,t393, t491, t590, t774, t803, t1387, t1492, t2574	9/44	–
**CC80 (55; 16)**	ST80	IVc, IVnt	t044, t067, t131, t376	48/51	–
	ST80	MSSA	t044, t131, t934	4/4	–
**CC30 (46; 14)**	ST30, ST1456	IVc	t019, t1133, t7709	8/9	–
	ST30, ST34 ST1472, ST1833	MSSA	t012, t018, t021, t032, t037, t122, t136, t238,t318, t342, t433, t665, t710, t871, t2509, t4275	6/37	–
**CC59 (31; 9)**	ST59, ST338, ST375	IVa, V	t172, t216, t437, t441	20/27	5/27
	ST59, ST375	MSSA	t216, t316, t437	–	
**CC121 (18; 6)**	ST121	MSSA	t159, t2019, t272, t284, t435, t1114, t4685,t6031, t645, t6870, t6872	7/18	–
**CC7 (18; 5)**	ST7	IVa, V	t091	1/2	–
	ST7	MSSA	t091, t796, t7710	–	–
**CC97 (10; 3)**	ST97	IVa	t267	–	–
	ST97	MSSA	t1965, t267, t3380, t359	–	–
**CC25 (8; 2)**	ST25, ST1595	MSSA	t078, t081, t280, t2909, t3644, t9040	1/8	–
**S93 (3; 1)**	ST93	IVa	t202, t1819	3/3	–

NT- non typable.

Clonal complexes (CC) were assigned by applying the e-BURST algorithm to the data obtained in this study and comparing it with the entire MLST database (www.mlst.net). This analysis was performed on October 20th 2011.

Among the 61 MSSA that belonged to CA epidemic clones and were collected from persons with no recent hospital contact, the most frequent clone was identified as ST30 (12 isolates), followed by ST121 (11 isolates), but ten additional STs were also identified: ST1, ST7, ST8 ST15, ST25, ST59, ST80, ST97, ST1472 and ST1595 (a SLV of ST25). Overall a higher genetic diversity was observed among MSSA than among MRSA [SID =  0.977 (0.966–0.989)].

### Population Structure of *S. aureus* Isolated from the Community-onset Setting

A total of 322 isolates (57%) out of the 568 analyzed in this study were collected in the first 48 hours of hospitalization from patients with at least one risk factor for hospital contact, and were considered as community-onset *S. aureus*.

Of the 322 isolates, 193 (60%) belonged or were related with community-associated epidemic clones, 85 (26%) were related to hospital-associated epidemic clones and 42 (13%) were sporadic. In addition, we identified two isolates (1%) that belonged to LA-MRSA epidemic clones (ST398-IVa and ST398-VII) (Table 1).

From the 85 *S. aureus* collected in the community-onset setting but belonging to HA epidemic clones, 43 were MRSA and 42 were MSSA. The most frequent clones among MRSA were E-MRSA15 (ST22-IVh, 24 isolates, 56%), followed by the Pediatric (ST5-IV/VI, seven isolates, 16%) and EMRSA 3 (ST5-I, five isolates, 12%). In addition, we also found the Berlin clone (ST45-IV, four isolates), New York/Japan (two isolates) and USA500 (ST8-IV, one isolate). The following clones were found among MSSA: ST45 (20 isolates, 48%), ST5 (15 isolates, 36%) and ST22 (seven isolates, 16%).

Of the 193 isolates belonging or related to community-associated epidemic clones, 96 (50%) were MRSA. Overall, the population structure of CA-MRSA and CO-MRSA isolates was similar. As observed for CA-MRSA isolates, the most frequent clones among CO-MRSA were USA300-related and the European related clone. Other clones that were common in the two populations were the Taiwan clone (ST59-IVa), the Southwest Pacific-related clone (ST30-IV), the Queensland clone (ST93-IVa) and ST97-IVa. However, we also found clones that were only prevalent among the onset isolate collection, like a SLV of Taiwan-clone, ST338-V, ST7-IVa, ST7-V, ST1-V and ST1835-V (Table 3). Overall CO-MRSA showed a genetic diversity similar to that observed for CA-MRSA isolates as defined by the combination of *spa* types with the presence of PVL and ACME [SID = 0.897 (0.863–0. 931)].

**Table 3:** Distribution of the 338 MRSA and MSSA isolates belonging to epidemic CA-MRSA clones or related in the community and community-onset settings.

Community-associated epidemic clones and related variants (%)		
	MRSA (52%), 84 isolates	MSSA (48%), 61 isolates
Community-associated	USA300 and related (35%)	ST30 (20%)
	European and related (33%)	ST121 (18%)
	Taiwan and related (13%)	ST7 (13%)
	ST772-V (6%)	ST15 (13%)
	ST1-IVa (4%)	ST97 (8%)
	ST97-IVa (3%)	ST80 (7%)
	USA700 (1%)	ST59 (5%)
	ST188-IVa(1%)	ST25 (5%)
	ST15-IVa (1%)	ST1 (3%)
	ST30-IVa (1%)	ST8 (3%)
	ST30-IVc (1%)	ST1595 (3%)
	ST93-IVa (1%)	ST1472 (2%)
	**MRSA (50%), 96 isolates**	**MSSA (50%), 97 isolates**
Community-onset	USA300 and related (42%)	ST30 (22%)
	European and related (24%)	ST15 (21%)
	Taiwan and related (17%)	ST1 (14%)
	Southwest Pacific (5%)	ST8 (11%)
	USA700 (2%)	ST7 (8%)
	Queensland (2%)	ST121 (7%)
	ST1456-IVc (2%)	ST72 (7%)
	ST772-NT(1%)	ST25 (3%)
	ST7-IVa (1%)	ST97 (2%)
	ST7-V (1%)	ST34 (2%)
	ST1-V (1%)	ST59 (1%)
	ST1835-V (1%)	ST1833 (1%)
	ST97-IVa (1%)	ST1867 (1%)

Concerning the 97 CO-MSSA isolates, we observed that its population structure differed from that found for the group of community-associated isolates analyzed in this study. The prevalent clones among CO isolates (ST30 and related STs and ST15 and related STs) were different from those identified among CA isolates. Moreover, we observed that although all clonal types found among CA isolates were also present in the CO collection, there were some clonal types that in our study were specific of the onset setting, namely ST34, ST72, ST1833 and ST1867 (Table 3). However, this was not translated into a higher genetic diversity [SID = 0.970 (0.957–0.982)].

### Clonal Variation and Genetic Diversity Along Time

Although sampling was performed between 2000–2009, the genetic diversity observed among the two settings was consistent throughout the entire time-frame analyzed (Information S1). A slight increase in genetic diversity was observed, particularly from the first period (2000–2001) to the second one (2002–2003), though this increase is not statistically significant.

Moreover, we observed that the number of isolates belonging to each clone varied along time. Whereas the European clone was the most frequent between 2004 and 2006, the frequency of USA300 and Taiwan clones increased in the last two years, when these clones became prevalent (Information S1).

### Distribution of PVL, ACME and SCC*mec* Among *S. aureus* Clones

We found an unexpectedly high prevalence of PVL among community-associated isolates (77/145, 53%) and community-onset isolates (98/193, 51%). Interestingly, PVL was not limited to specific genetic backgrounds or clones, but otherwise was found to be disseminated among all clonal complexes identified among CA epidemic clones with the exception of CC97.

The great majority of the PVL-positive isolates were MRSA (83%, 146 out of 175 isolates) and belonged to USA300 clone and its variants, the European clone and its variants and the Taiwan clone and related clones. Moreover, PVL genes were identified in eight additional clones (ST1-IVa, ST7-IVa, ST30-IVc, ST30-IVa, ST1456-IVc, ST772-V, USA700-ST72-IVc and Queensland-ST93-IVa) (Table 2 and Information S1). Regarding the 29 MSSA isolates, PVL genes were disseminated among a total of nine STs, namely ST1, ST8, ST15, ST25, ST30, ST72, ST80, ST121 and ST1472.

Like for PVL, the distribution of ACME among community-associated isolates (21/145, 15%) was similar to that found among community-onset isolates (25/193, 13%) and was mostly associated to MRSA (45 out of 46 isolates). However, unlike PVL, ACME distribution was limited only to two clonal complexes (CC8 and CC59).

ACME-positive isolates belonged or were related to USA300, the Taiwan clone and USA700. ACME-I was identified exclusively among USA300-related isolates, while ACME-II, containing only the *arc* operon, was identified in isolates of the other two epidemic clones.

SCC*mec* typing showed that the great majority of isolates (73 CA and 73 CO, 81%) carried SCC*mec* IV, though SCC*mec* V (28 isolates, 16%) and VI (four isolates, 2%) were also detected. In addition, two isolates (1%) presented a non-typable SCC*mec*. The most common SCC*mec* IV subtype was SCC*mec* IVc (78 isolates, 53%), followed by SCC*mec* IVa (62 isolates, 42%). SCC*mec* IVd and IVg were rare (one isolate each) and four isolates carried a non-subtypable SCC*mec* IV.

### Antibiotic Resistance

As expected, we found a very low rate of antibiotic multi-resistance in CA-MRSA from both settings, 11.6% (21 isolates out of 181). The results are summarized in Table 4. Multi-resistance occurred exclusively in USA300 clone and its variants, in the European clone, Taiwan and related clones and a ST772-V isolate. Two isolates resistant to four classes of antimicrobial agents were identified and belonged to clones ST8-IVc, t024, PVL+ and ST772-V, t1387, PVL+. Regarding MSSA, antibiotic resistance rates were extremely low. In fact, only 19.9% (36 isolates out of 181) of the isolates were resistant to at least one class of antibiotics and a single isolate was identified as multi-drug resistant (0.5%, ST121, resistant to ciprofloxacin, erythromycin, clindamycin and tetracycline).

**Table 4:** Molecular characteristics of multidrug-resistant MRSA isolates belonging to epidemic community-associated clones collected in the community and community onset settings.

Antibiotic Resistance	Genetic background (no isolates)
Beta-lactams, Fus acid, Tet	ST80-IVc, t044, PVL+, ACME - (2)
	ST80-IVc, t131, PVL+, ACME- (1)
	ST8-IVc, t024, PVL+, ACME- (1)
	ST375-IVa, t172, PVL-, ACME- (1)
	ST59-V, t437, PVL+, ACME II (1)
	ST59-V, t437, PVL+, ACME – (1)
Beta-lactams, Fus acid, Ery	ST80-IVc, t044, PVL+, ACME - (3)
	ST80-IVnt, t044, PVL+, ACME – (1)
	ST8-IVc, t024, PVL+, ACME – (1)
Beta-lactams, Cipro, Ery	ST8-IVa, t008, PVL+, ACME I (3)
	ST8-IVc, t008, PVL+, ACME – (1)
Beta-lactams, Tet, Ery	ST8-IVc, t024, PVL+, ACME- (1)
	ST80-IVc, t044, PVL+, ACME- (1)
	ST59-IVa, t437, PVL-, ACME II (1)
Beta-lactams, Ery, Clind, Tet, Fus acid	ST8-IVc, t024, PVL+, ACME- (1)
Beta-lactams, Ery, Tet, Gent	ST772-V, t1387, PVL+, ACME- (1)

Cipro – ciprofloxacin; Clind – clindamycin; Ery – erythromycin; Fus acid – fusidic acid; Tet – tetracycline; Gent – gentamicin.

### Geographic Distribution of CA-*S. aureus* in Europe

**Figure 1:**
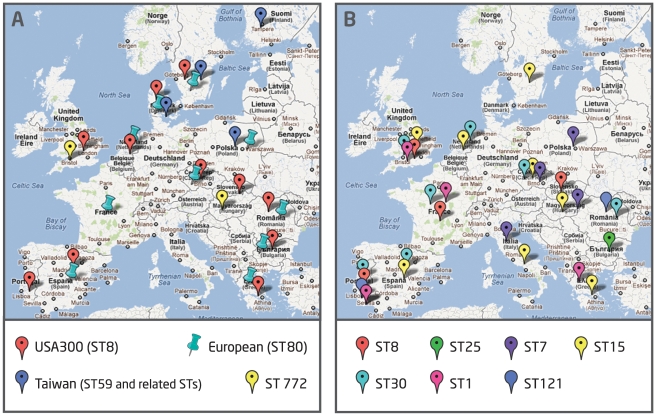
Prevalence of MRSA and MSSA community-associated clones in Europe. Distribution of the most prevalent MRSA and MSSA community-associated epidemic and related clones in 16 of the most populous European countries. Each color represents a different clone and related clonal lineages. A –MRSA; B –MSSA.

Overall, a high genetic diversity was found among community-associated and community-onset *S. aureus* isolates belonging to CA epidemic clones (Figure 1). However, some asymmetry was observed in what respects to the number of different clonal types found in each country. Whereas in The Netherlands as many as ten different clonal types were identified among 22 isolates (ST80-IVc, ST772-V, ST8-IVa, ST8-IVnt, ST7-V, ST30-IVc, ST398-IVc, ST398-VII, ST93-IVa, ST97-IVa), in Poland only three types among 16 isolates were found (ST338-V, ST80-IVc, ST7-IVa) (Information S1).

In spite of the genetic diversity observed, all the clonal types identified were disseminated in more than one country and neighboring countries shared more clonal types than distant countries. The most epidemic clonal type among MRSA was the European clone (ST80-IVc) that was found in eleven different countries, followed by USA300 (ST8-IVa) that was recovered in nine countries.

Interestingly, some specificity was observed in what respects the distribution of the most prevalent clones. Whereas USA300, the European or related clones were the most prevalent in Southern and Central European countries, in Northern Europe (Finland, Sweden and Poland) the most prevalent MRSA clonal type was related with the Taiwan clone (Figure 1 and Information S1).

Regarding MSSA, the most disseminated clone was ST15 and related clonal types that were identified in eleven different countries, followed by ST121 and ST30 and its derivatives that were identified in nine countries each.

### CA-MRSA Origin

**Figure 2:**
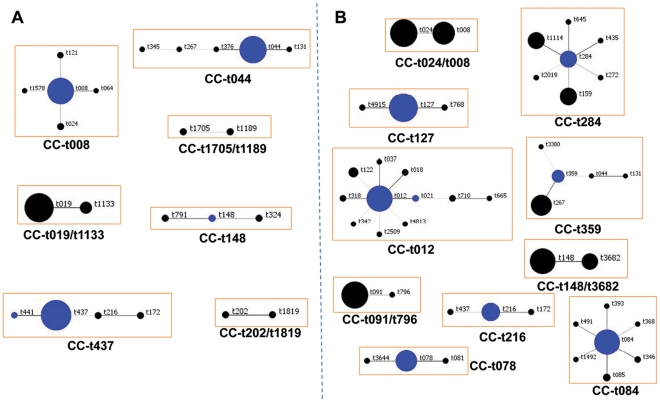
Analysis of *spa* typing data obtained for MRSA and MSSA isolates. *spa* typing data from isolates belonging to epidemic and related CA-MRSA clones and sporadic isolates collected in the community and community-onset settings was analyzed by BURP (http://spaserver.ridom.de/, StaphType software v. 1.5, Ridom GmbH, Würzburg, Germany). Each *spa* type identified is depicted with circles. Related *spa* types are connected with a black line; resultant clonal complexes are depicted inside orange boxes. The predicted founder of each clonal complex is indicated in blue and in a larger circle. The size of the circles is proportional to the frequency of the *spa* type in the population. Each clonal complex (CC) is defined by the predicted founder *spa* type or by the *spa* types it contains. A –MRSA; B – MSSA.

In order to understand the origin of the CA-MRSA clones presently circulating in Europe, we analyzed the relatedness of *spa* types identified in MSSA and MRSA isolates belonging to the same clonal lineage by BURP analysis of the *spa* types (Figure 2).

BURP analysis showed that MRSA and MSSA isolates belonging to ST8 (CC-t008, CC-t024, CC-1705/1189), ST72 (CC-t148, CC-t148/t3682) and ST59 (CC-t437, CC-t216) or its SLVs (Figure 2) shared the same or related *spa* types, suggesting that CA-MRSA and CA-MSSA isolates belonging to these STs were closely related.

On the other hand, for MRSA and MSSA isolates from ST30 (CC-t012) and ST93 (CC-t202/t1819) genetic clones no common or related *spa* types were found. Regarding isolates belonging to the European clone, no conclusions could be drawn since only a small number of MSSA isolates belonging to this specific genetic background was found in our collection.

## Discussion

The recent establishment of CA-MRSA as a leading cause of infections in healthy individuals is a matter of great concern. Previous studies that have addressed the epidemiology of this pathogen indicate that most of the infections are caused by a limited number of specific clones that in addition seem to be geographically restricted [19], [47], [50]. In this study we report for the first time the population structure of CA-MRSA in Europe and describe the very high level of genetic diversity and epidemicity of CA-MRSA clones on this continent.

As many as ten different CA-MRSA clones in a single country were found. Moreover, several different variants of already described clonal types were identified, differing in the nucleotide sequence of housekeeping genes or in the SCC elements content and virulence genes; several new or rare CA-MRSA clonal types (ST772-V, ST7-V, ST188-IVa, ST15-IVa, ST375-IVa and ST338-V) were identified. Similar results were described in studies conducted in some European countries [51], [52], [53], but it was never observed at a global scale. The reason for the large genetic diversity observed, in contrast to the situation of a single epidemic CA-MRSA clone described in USA, is not obvious. We suggest that the multiplicity of cultural and social behaviors and habits inherent to each European country, namely frequency and destination of travel, different hygienic habits, infection control measures and antibiotic prescription and consumption policies may have shaped the population structure of CA-MRSA on this continent. Moreover, the geographic proximity of the countries and the travel habits resultant of tourism and business can also have had a role on the dissemination of clonal types among the different European countries.

Interestingly, the most frequent clone detected in the collection was USA300 or related clones, which contrasts sharply with previous studies in Europe in which the European clone was found to be predominant [19]. This represents a changing trend in the epidemiology of CA-MRSA in Europe. The USA300 clone is well adapted to the community environment; it carries ACME and SCC*mec* IV, which are believed to confer a higher fitness to the clone [45], [46]. Around 33% of all ST8 isolates had all the characteristics of the USA300 clone, suggesting that this clone was imported and is becoming well established in Europe. However, the great majority of ST8 isolates was highly related with USA300, but lacked one of its typical molecular characteristics or had others instead (for instance, the presence of SCC*mec* V or the absence of PVL). We were able to detect as many as six different SCC*mec* (sub) types and nine *spa* types associated to ST8, indicating that different sub clones exist in the population structure of this ST. The high genetic diversity that we found among ST8 isolates was already observed by others [54], [55], [56]. The origin of such variants is unknown. They may have derived from the epidemic USA300 strain imported from the USA and evolved rapidly in Europe in order to adapt to different selective pressures, namely by acquiring/losing virulence factors as PVL or antibiotic resistance determinants as SCC*mec*. Alternatively, they may have diverged from a USA300 MSSA ancestor early in time and established as a different clone in Europe by the acquisition of an SCC*mec* element. The finding in our study of MSSA and MRSA isolates belonging to the same *spa* type that is different from t008 supports the existence of a European ST8 clone. However, only the whole genome sequence of these strains will clarify this hypothesis.

Besides ST8, we also found MRSA and MSSA isolates belonging to ST72 and ST59 that shared the same *spa* types, which suggests that these specific genetic backgrounds could have also emerged recently in Europe by the acquisition of SCC*mec* in already established MSSA clones. On the contrary, for ST30 and ST93 or related clones no common or related *spa* types were found between MRSA and MSSA, suggesting that CA-MRSA belonging to these clones were probably created outside Europe and imported later.

The continuous evolution of CA-MRSA in Europe could explain the high level of genetic diversity of SCC*mec* found among CA-MRSA belonging to the same ST: by emerging in Europe, these isolates could have acquired the most common SCC*mec* in each specific location. A similar proposition has been made by Nübel et al [57] to explain the diversity among ST5 MRSA. Although our proposition is plausible we cannot disregard the hypothesis that the different genetic backgrounds were imported as MRSA and later lost the SCC*mec* in Europe in the absence of selective pressure.

The collection analyzed in this study was not part of a structured survey with pre-defined criteria of isolate collection. It was a convenience sample composed of isolates collected within 48 hours of hospital admission. Consequently, the number of isolates obtained was not equal from country to country and the timeframe of isolation spanned almost 10 years. The inclusion of a low number of isolates in some countries might have provided an erroneous picture of the local epidemiology that wrongly influenced the overall genetic diversity described in this study. Moreover, due to the long timeframe of sampling, the genetic diversity observed in this study might be inflated, not reflecting absolutely the present reality in Europe.

We found an unexpectedly high prevalence of PVL in our collection (52%) and a high level of dissemination among *S. aureus* CA-epidemic clones. To our knowledge, PVL was only rarely reported among *S. aureus* collected in Europe [58], [59] and was usually associated with the European clone [19]. However, there are studies that report an increasing frequency of this leukocidin in isolates collected in Europe [53], [60]. The data that resulted from our study suggest that PVL frequency in Europe may be increasing, not only due to the dissemination of PVL positive epidemic CA-MRSA clones, but also due to *de novo* acquisition of phage encoded PVL by different genetic backgrounds. Surveillance measures should be taken in order to detect these leukocidin-producing isolates, since several studies have indicated a connection between the existence of PVL and the outcome of the disease [11], [61], [62], [63], [64].

In contrast we found a low frequency of ACME (14%) mainly associated with the USA300 clone (ST8-IVa). Noteworthy, we found some isolates related with the Taiwan clone (ST59-V) and USA700 (ST72-IV) carrying ACME-II. This fact is particularly relevant if one take into consideration that acquisition of ACME by already epidemic *S. aureus* clones may increase their capacity of dissemination [46].

The CA-MRSA population structure in the community and community onset settings were almost identical in what regards to distribution of the most prevalent clones, however certain clones were only identified among the onset-population. The results suggest that a high number of different CA-MRSA clonal types are at risk of entering hospital environment through the community-onset population.

In this study we identified a tremendously high level of genetic diversity among CA-MRSA in Europe as well as a high frequency of PVL-positive isolates. This scenario poses an unprecedented challenge not only to diagnostic but also to infection control. The fast CA-MRSA evolution in Europe demands a continuous surveillance as a means to help local health-care providers in designing strategies to detect and control CA-MRSA.

## Supporting Information

Information S1 **Additional molecular and epidemiological information.**
(DOCX)
