# Supplementary material for: High Genetic Diversity among Community-Associated Staphylococcus aureus in Europe: Results from a Multicenter Study
Source: PLoS One. 2012 Apr 27;7(4):e34768. doi: 10.1371/journal.pone.0034768 (PMC3338755; doi:10.1371/journal.pone.0034768)
Supplement: Information S1 — Additional molecular and epidemiological information. (DOCX) [file pone.0034768.s001.docx]

Suplemental table S 1. Molecular and epidemiological characteristics of the 338 CA and CO-*S. aureus* isolates analyzed in this study that belonged to epidemic CA clones.

| **Country (no of isolates)** | **Sampling geographic regions** | **Year of isolation** | **Clonal types^a^ (no of isolates)** |
| --- | --- | --- | --- |
| Bulgaria (16) | Sofia  Lovetch  Shumen  Sliven  Vratza | 2000-2005 | ST8- IVa – t008 – PVL+; ACME I (7)  ST8-NT- t008 – PVL+; ACME III (1)  ST80-IVc-t044-PVL+; ACME- (1)  ST1595-MS-t078-PVL-; ACME-(2)  ST15-MS-t085-PVL-; ACME- (1)  ST25-MS- t078 – PVL-; ACME – (1)  ST25-MS- t2909 – PVL-; ACME – (1)  ST97-MS-t267-PVL-; ACME- (1)  ST121-MS-t435-PVL+; ACME- (1) |
| Czech Republic (43) | České Budějovice  Pardubice  Klatovy  Kladno  Trutnov  Plzeň  Kolín  Prague  Opava  Hradec Králové  Jihlava  Mělník  Příbram | 2002-2005  2008-2009 | ST8- IVa – t008 – PVL+ ; ACME I (6)  ST8- IVnt – t008 – PVL+ ; ACME - (2)  ST8- V – t024 – PVL- ; ACME - (2)  ST8- IVa – t008 – PVL+ ; ACME - (1)  ST8- IVc – t008 – PVL- ; ACME - (1)  ST8- IVd – t064 – PVL- ; ACME - (1)  ST80-IVc-t044-PVL+; ACME- (4)  ST80-IVc-t131-PVL+; ACME- (1)  ST30-IVc-t019-PVL+ ; ACME- (1)  ST72-IVa-t791-PVL+ ; ACME-(1)  ST93-IVa-t1819-PVL+ ; ACME- (1)  ST7-MS-t091-PVL- ; ACME- (4)  ST30-MS-t122-PVL- ; ACME-(3)  ST1-MS-t184-PVL- ; ACME- (2)  ST15-MS-t084-PVL- ; ACME-(2)  ST72-MS-t3682-PVL- ; ACME-(2)  ST1-MS-t127-PVL- ; ACME- (1)  ST8- MS – t008 – PVL+ ; ACME - (1)  ST8- MS – t024 – PVL- ; ACME - (1)  ST15-MS-t2574-PVL- ; ACME-(1)  ST15-MS-t491-PVL- ; ACME-(1)  ST15-MS-t346-PVL- ; ACME-(1)  ST72-MS-t148-PVL- ; ACME-(1)  ST72-MS-t5986-PVL- ; ACME-(1)  ST121-MS-t6031-PVL-; ACME- (1) |
| Denmark (23) | Not available | 2009 | ST8- IVa – t008 – PVL+ ; ACME I (7)  ST30-IVc-t019-PVL+ ; ACME-(2)  ST59-IVa-t216-PVL+ ; ACME-(2)  ST59-IVa-t437-PVL- ; ACME-(2)  ST80-IVc-t044-PVL+; ACME- (2)  ST80-IVc-t376-PVL+; ACME- (2)  ST97-IVa-t267-PVL- ; ACME- (2)  ST1456-IVc-t1133-PVL+ ; ACME -(2)  ST1-V-t127-PVL- ; ACME-(1)  ST1835-V-t127-PVL- ; ACME- (1) |
| Finland (11) | Kuusankoski  Joensuu  Oulu  Espoo  Äänekoski  Kuopio  Vantaa  Turku  Lahti  Helsinki  Nivala  Seinäjoki  Tampere  Mikkeli | 2004-2006 | ST375-IVa-t172-PVL- ; ACME- (3)  ST1-IVa-t4915-PVL+ ; ACME-(1)  ST1-IVa-t127-PVL+ ; ACME-(1)  ST8- IVa – t008 – PVL+ ; ACME I (1)  ST30-IVa-t019-PVL- ; ACME-(1)  ST30-IVc-t7709-PVL- ; ACME-(1)  ST80-IVc-t131-PVL+ ; ACME-(1)  ST93-IVa-t202-PVL+ ; ACME- (1)  ST772-V-t1387-PVL+; ACME-(1) |
| France (22) | Lyon | 2005 | ST80-IVc-t044-PVL+; ACME- (4)  ST1-MS-t127-PVL+; ACME- (4)  ST1-MS-t590-PVL+ ; ACME- (2)  ST8-MS-t008-PVL- ; ACME- (2)  ST15-MS-t084-PVL+ ; ACME- (2)  ST30-MS-t012-PVL-; ACME-(2)  ST30-MS-t433-PVL+ ; ACME-(2)  ST121-MS-t4685-PVL+; ACME-(2)  ST8-MS-t024-PVL- ; ACME- (1)  ST121-MS-t645-PVL- ; ACME-(1) |
| Greece (30) | Patras | 2005 | ST80-IVc-t044-PVL+; ACME- (19)  ST8- IVa – t008 – PVL- ; ACME - (1)  ST80-IVnt-t044-PVL+; ACME- (1)  ST1-MS-t127-PVL-; ACME- (2)  ST15-MS-t084-PVL- ; ACME- (2)  ST7-MS-t796-PVL-; ACME- (1)  ST25-MS-t9040-PVL-;ACME-(1)  ST30-MS-t012-PVL- ; ACME-(1)  ST80-MS-t044-PVL+; ACME- (1)  ST121-MS-t2019-PVL- ; ACME- (1) |
| Hungary (13) | Budapest | 2009 | ST15-IVa-t084-PVL- ; ACME- (1)  ST7-MS-t091-PVL- ; ACME-(5)  ST15-MS-t084-PVL- ; ACME- (2)  ST7-MS-t7710-PVL- ; ACME-(1)  ST15-MS-t1492-PVL- ; ACME- (1)  ST59-MS-t216-PVL- ; ACME-(1)  ST59-MS-t316-PVL- ; ACME-(1)  ST97-MS-t267-PVL- ; ACME-(1) |
| Italy (3) | Rome | 2009 | ST7-MS-t091-PVL- ; ACME-(1)  ST15-MS-t084-PVL- ; ACME- (1)  ST97-MS-t3380-PVL- ; ACME-(1) |
| The Netherlands (37) | Eindhoven  Geldrop  Veldhoven  Deurne  Hapert  Helmond  Oirschot | 2006-2009 | ST80-IVc-t044-PVL+; ACME- (7)  ST8- IVa – t008 – PVL+; ACME I (4)  ST772-V-t657-PVL+; ACME- (4)  ST7-V-t091-PVL- ; ACME-(1)  ST8- IVnt – t008 – PVL- ; ACME II(1)  ST30-IVc-t019-PVL+ ; ACME- (1)  ST93-IVa-t202-PVL+ ; ACME-(1)  ST97-IVa-NT-PVL- ; ACME-(1)  ST30-MS-t012-PVL- ; ACME- (3)  ST80-MS-t934-PVL+; ACME- (3)  ST15-MS-t084-PVL- ; ACME- (2)  ST34-MS-t136-PVL- ; ACME- (2)  ST97-MS-t359-PVL-; ACME- (2)  ST7-MS-t091-PVL- ; ACME-(1)  ST15-MS-t085-PVL- ; ACME- (1)  ST30-MS-t318-PVL+ ; ACME-(1)  ST59-MS-t216-PVL- ; ACME- (1)  ST121-MS-t6870-PVL+ ; ACME- (1) |
| Poland (17) | Kołobrzeg  Warszawa  Gorlicac  Katowice  Maków Mazowiecki  Kościan | 2008-2010 | ST338-V-t437-PVL+; ACME-(13)  ST338-V-t441-PVL+; ACME-(1)  ST80-IVc-t044-PVL+; ACME- (1)  ST7-IVa-t091-PVL+ ; ACME-(1)  ST7-MS-t091-PVL- ; ACME-(1) |
| Portugal (22) | Portimão  Cascais  Braga  Coimbra  Lisboa  Santo Tirso  Oeiras | 2006  2008-2009 | ST8-VI-t008-PVL- ; ACME-(2)  ST8- IVa – t008 – PVL+ ; ACME I (1)  ST72-IVc-t148-PVL+ ; ACME-(1)  ST931-VI-t008-PVL+ ; ACME II (1)  ST939-IVa-t324-PVL+ ; ACME- (1)  ST30-MS-t012-PVL- ; ACME-(4)  ST1-MS-t127-PVL- ; ACME-(2)  ST8-MS-t008-PVL- ; ACME-(1)  ST8-MS-t024-PVL- ; ACME-(1)  ST25-MS-t280-PVL- ; ACME-(1)  ST25-MS-t3644-PVL+ ; ACME-(1)  ST30-MS-t342-PVL- ; ACME-(1)  ST72-MS-t148-PVL+ ; ACME-(1)  ST72-MS-t148-PVL- ; ACME II(1)  ST72-MS-t126-PVL+ ; ACME-(1)  ST121-MS-t159-PVL- ; ACME-(1)  ST121-MS-t284-PVL+ ; ACME-(1) |
| Romania (20) | Bucharest | 2005  2009 | ST1-IVa-t1381-PVL- ; ACME- (1)  ST8- IVa – t008 – PVL+; ACME I (1)  ST8- IVa – t008 – PVL- ; ACME I (1)  ST8- IVa – t008 – PVL- ; ACME II (1)  ST8- IVa – t5160 – PVL- ; ACME II (1)  ST80-IVc-t067-PVL- ;ACME –(1)  ST121-MS-t645-PVL- ; ACME-(4)  ST30-MS-t710-PVL- ; ACME-(2)  ST30-MS-t018-PVL- ; ACME-(2)  ST30-MS-t021-PVL+ ; ACME-(2)  ST121-MS-t1114-PVL- ; ACME-(2)  ST121-MS-t284-PVL+ ; ACME-(1)  ST7-MS-t091-PVL- ; ACME-(1) |
| Slovakia (5) | Nitra | 2009 | ST8- IVa – t008 – PVL+; ACME I (1)  ST8- MS – t024 – PVL; ACME - (4) |
| Spain (37) | Madrid  Barcelona | 2002-2005  2007-2009 | ST8- IVc – t008 – PVL+; ACME - (9)  ST8- IVc – t024 – PVL+ ; ACME -(3)  ST8- IVa – t008 – PVL+ ; ACME I (2)  ST8- IVc – t121 – PVL+ ; ACME - (2)  ST8- IVc – t1189 – PVL+; ACME - (1)  ST8- VI – t008 – PVL+; ACME - (1)  ST8- IVa – t121 – PVL+ ;ACME I (1)  ST8- IVa – t121 – PVL+ ; ACME II (1)  ST80-IVc-t067-PVL- ; ACME-(2)  ST30-IVc-t019-PVL+; ACME-(1)  ST30-MS-t012-PVL-; ACME-(4)  ST30-MS-t032-PVL-; ACME-(1)  ST30-MS-t238-PVL-; ACME-(1)  ST30-MS-t2509-PVL- ; ACME-(1)  ST30-MS-t4275-PVL- ; ACME-(1)  ST30-MS-t871-PVL- ; ACME-(1)  ST1-MS-t121-PVL- ; ACME-(1)  ST15-MS-t368-PVL- ; ACME-(1)  ST15-MS-t393-PVL- ; ACME-(1)  ST121-MS-t272-PVL+; ACME- (1)  ST1472-MS-t665-PVL+ ; ACME-(1) |
| Sweden (22) | Lund  Helsinborg | 2006  2008-2009 | ST80-IVc-t044-PVL+ ; ACME-(5)  ST59-V-t437-PVL+; ACME II (3)  ST59-IVa-t437-PVL-; ACME II (2)  ST59-V-t437-PVL+; ACME - (1)  ST8- IVa – t008 – PVL+; ACME II (1)  ST8- IVa – t1578 – PVL+ ; ACME I (1)  ST8- IVc – t024 – PVL+ ; ACME - (1)  ST72-IVc-t664-PVL- ; ACME-(1)  ST188-IVa-t189-PVL- - ACME-(1)  ST7-MS-t091-PVL- ; ACME-(1)  ST15-MS-t084-PVL- ; ACME-(2)  ST15-MS-t774-PVL- ; ACME-(1)  ST15-MS-t085-PVL+; ACME-(1)  ST121-MS-t6872-PVL- ; ACME-(1) |
| United Kingdom (17) | Birmingham | 2009 | ST8- IVg – t1705 – PVL-; ACME - (1)  ST772- NT – t345 – PVL+ ; ACME - (1)  ST1-MS-t273-PVL- ; ACME-(2)  ST8- MS – t008 – PVL- ; ACME - (1)  ST8- MS – t024 – PVL- ; ACME - (1)  ST15- MS – t084 – PVL- ; ACME - (2)  ST15- MS – t346 – PVL- ; ACME - (1)  ST15- MS – t803 – PVL- ; ACME - (1)  ST25- MS – t081 – PVL- ; ACME - (1)  ST30-MS-t122-PVL- ; ACME-(1)  ST59- MS – t437 – PVL- ; ACME - (1)  ST97- MS – t359 – PVL- ; ACME - (1)  ST97- MS – t1965 – PVL-; ACME - (1)  ST1833- MS – t037 – PVL -; ACME - (1)  ST1867- MS – t084 – PVL- ; ACME - (1) |

^a^Clonal types are defined by sequence type-SCC*mec* type (when present; MS:methicillin susceptible) – *spa* type - PVL presence(+:positive; -:negative); ACME presence/type (I/II/III; -negative)

**Supplemental table S2.** Degree of genetic diversity observed in the population of isolates collected in specific periods of time. Simpson’s Index of Diversity (SID) with a 95% confidence interval is shown.

| **Year blocks** | **Simpson’s Index of Diversity (SID), 95% CI** |
| --- | --- |
| 2000-2001 | 0.644 (0.363-0.926) |
| 2002-2003 | 0.872 (0.768-0.976 |
| 2004-2005 | 0.936 (0.899-0.973) |
| 2006-2008 | 0.953 (0.913-0.994) |
| 2009-2010 | 0.966 (0.956-0.977) |

**Supplemental figure 1.** Distribution of the most frequent clones (number of isolates) in European countries per year of isolation.
